# Glucose-Induced Down Regulation of Thiamine Transporters in the Kidney Proximal Tubular Epithelium Produces Thiamine Insufficiency in Diabetes

**DOI:** 10.1371/journal.pone.0053175

**Published:** 2012-12-28

**Authors:** James R. Larkin, Fang Zhang, Lisa Godfrey, Guerman Molostvov, Daniel Zehnder, Naila Rabbani, Paul J. Thornalley

**Affiliations:** Warwick Medical School, Clinical Sciences Research Laboratories, University of Warwick, University Hospital, Coventry, United Kingdom; Virginia Commonwealth University, United States of America

## Abstract

Increased renal clearance of thiamine (vitamin B_1_) occurs in experimental and clinical diabetes producing thiamine insufficiency mediated by impaired tubular re-uptake and linked to the development of diabetic nephropathy. We studied the mechanism of impaired renal re-uptake of thiamine in diabetes. Expression of thiamine transporter proteins THTR-1 and THTR-2 in normal human kidney sections examined by immunohistochemistry showed intense polarised staining of the apical, luminal membranes in proximal tubules for THTR-1 and THTR-2 of the cortex and uniform, diffuse staining throughout cells of the collecting duct for THTR-1 and THTR-2 of the medulla. Human primary proximal tubule epithelial cells were incubated with low and high glucose concentration, 5 and 26 mmol/l, respectively. In high glucose concentration there was decreased expression of THTR-1 and THTR-2 (transporter mRNA: −76% and −53% respectively, *p*<0.001; transporter protein −77% and −83% respectively, *p*<0.05), concomitant with decreased expression of transcription factor specificity protein-1. High glucose concentration also produced a 37% decrease in apical to basolateral transport of thiamine transport across cell monolayers. Intensification of glycemic control corrected increased fractional excretion of thiamine in experimental diabetes. We conclude that glucose-induced decreased expression of thiamine transporters in the tubular epithelium may mediate renal mishandling of thiamine in diabetes. This is a novel mechanism of thiamine insufficiency linked to diabetic nephropathy.

## Introduction

Renal mishandling of thiamine (vitamin B_1_) in diabetes has recently emerged as a factor linked to the development of diabetic nephropathy [1], [2]. Patients with type 1 or type 2 diabetes had increased renal clearance and fractional excretion of thiamine compared to healthy people [3], [4]. This was not linked to microalbuminuria although fractional excretion of thiamine at onset of microalbuminuria was a risk predictor of subsequent decline in renal function [5]. Increased renal clearance and fractional excretion of thiamine in clinical diabetes were not linked to dietary intake of the vitamin, as judged from lack of correlation with urinary thiamine excretion [4] – an indicator of dietary intake of thiamine [6]. Rather, in both clinical and experimental diabetes increased renal clearance of thiamine was linked to decreased reuptake of thiamine from the glomerular filtrate [3], [7], and in clinical diabetes to glycemic control - reflected in a positive correlation to glycated hemoglobin HbA_1c_ [4].

Oral thiamine supplements prevented the development of incipient nephropathy in streptozotocin (STZ)-induced diabetic rats [7]. Two clinical studies evaluating the use of thiamine supplements in type 2 diabetic patients with microalbuminuria to counter the washout of the vitamin found that urinary albumin excretion was decreased and in some cases reverted to normoalbuminuria by thiamine supplementation [8], [9]. It seems likely that the benefits of thiamine supplementation are achieved by countering diabetes-induced renal washout of thiamine. The mechanism of decreased renal re-uptake of thiamine in diabetes is not known.

Thiamine is a water-soluble vitamin and essential micronutrient. It is converted to thiamine pyrophosphate (TPP) by thiamine pyrophosphokinase. TPP is co-factor of pyruvate dehydrogenase, α-ketoglutarate dehydrogenase and transketolase. Pyruvate dehydrogenase is essential for entry of pyruvate into the citric acid cycle and aerobic glycolysis and α-ketoglutarate dehydrogenase catalyses a step in the cycle. Transketolase has a key role in oxidative and non-oxidative branches of the pentose phosphate pathway and a key role in resisting vascular cell dysfunction associated with the development of diabetic nephropathy [10].

Thiamine is an organic cation and crosses cell membranes at normal physiological concentrations via specific, high affinity thiamine transporters. It is readily filtered by renal glomeruli and is reabsorbed from the glomerular filtrate into the renal venous circulation by thiamine transporters in the renal brush border membrane; influx is increased by an outwardly directed H^+^ gradient [11]. At high concentrations achieved by pharmaceutical dosing, thiamine may also cross cell membranes by non-saturable passive diffusion of the thiazolium ring-opened, unionized form [12]. There are two thiamine transporters present in human tissues, thiamine transporters-1 and -2 (THTR-1 and THTR-2), encoded by genes *SLC19A2* and *SLC19A3* respectively [13]. There is relatively high expression of these transporters in the kidney [14]–[16]. The K_M_ for thiamine transport by THTR-1 is 2.5 µM and by THTR-2 is 27 nM [14], [17]. Basal expression of THTR-1 and THTR-2 transporters is dependent on transcription factor specificity protein-1 (Sp1) [18], [19].

In this study, we investigated the location of THTR-1 and THTR-2 in the human kidney, studied the effect of high glucose concentration on the expression of thiamine transporters in human proximal tubular epithelial cells (hPTECs) and the human tubular epithelial HK-2 cell line *in vitro*, and the effect of intensive glycemic control of renal mishandling of thiamine in experimental diabetes.

## Materials and Methods

This study was carried out in strict accordance with the recommendations in the “Guide for the Care and Use of Laboratory Animals of the National Institutes of Health”. Animal studies were performed on UK Home Office project license no 40/3261. All efforts were made to minimize suffering.

The Coventry & Warwickshire Research Ethics Committee approved the project (reference number 10/H1211/36) to obtain human kidney samples and informed written consent was obtained from each patient before undergoing therapeutic nephrectomy.

### Materials

Antibodies used were: mouse monoclonal anti-aquaporin-1 peptide fragment antibody, ab9566 (AbCam, Cambridge, UK); rabbit polyclonal anti-THTR-1 and anti-THTR-2 antibodies, codes THTR12-A and THTR22-A, respectively (Alpha Diagnostic International San Antonio, TX, USA); and mouse polyclonal anti-uromodulin antibody (Abnova, Taipei City, Taiwan). The HK-2 cell line was purchased from American Type Culture Collection, Manassas, VA, USA.

### Isolation of human proximal tubule epithelial cells

Human proximal tubule epithelial cells (hPTECs) were isolated from human kidney samples. Decapsulated kidney cortex (*ca.* 1.5 g wet weight) from healthy human donors was diced finely on ice and digested in 100 ml PBS with 5 mmol/l CaCl_2_ and 0.1% (w/v) type II collagenase (Worthington Biochemical Corporation, Lakewood, NJ, USA) in a shaking water bath (37°C, 180 rev/min, 40 min). The digested cortex was passed through a 212 µm stainless steel sieve, removing undigested material. Cells and tubule fragments in the filtrate were collected by centrifugation (2 min, 125 g, 4°C), washed thrice and resuspended in ice-cold DMEM/F12 medium (5 mmol/l glucose and 4 nmol/l thiamine) supplemented with 1% penicillin-streptomycin, 20 mmol/l NaHCO_3_ and 15 mmol/l HEPES at pH 7.4. The washed cellular material was separated by density-gradient centrifugation on 31% Percoll/NaCl, osmolarity 300 mOsm (30 min, 30,000 g, 4°C). Density fractions were re-suspended in 37°C DMEM/F12 and supplemented with 2.5% fetal bovine serum, seeded in tissue culture flasks and cell composition characterized by immunostaining for aquaporin 1 and uromodulin.

### Immunohistochemistry

This was performed on paraffin-embedded sections of normal human kidney fixed onto glass slides and also cells in primary culture fixed with 4% formaldehyde in PBS overnight. Paraffin sections were dewaxed in xylene, 100% and 90% ethanol and water sequentially. All slides were subject to antigen retrieval (4.13 mmol/l tris base, 1.71 mmol/l EDTA, 1.24 mmol/l citric acid, NaOH to pH 7.8, 20 min, 120°C, 2 bar). The slides were stained with 3,3′-diaminobenzidine using primary antibody and Vectastain Universal Quick kit with avidin-biotin blocking kit (Vector Laboratories, Peterborough, UK). Nuclei were counterstained blue with Meyer's hematoxylin.

### Cell culture methods

The HK-2 cell line and primary human proximal tubular epithelial cells (hPTECs) were cultured in custom made DMEM/F12 medium with 5 mmol/l glucose and 4 nmol/l thiamine – a thiamine concentration typical of the lower quartile-median range of patients with diabetes [3], [8] - under an atmosphere of 5%CO_2_/95% air 37°C and 100% humidity. Cells were seeded into either 5 mmol/l or 26 mmol/l glucose medium with 4 nmol/l thiamine and cultured for five days before harvesting. Cells were passaged at ∼90% confluence using 0.25% (w/v) trypsin-EDTA solution.

### Real time qPCR

Total cellular RNA was extracted from frozen cell pellets using the Qiagen RNeasy mini kit (Crawley, UK). The reverse transcription step was performed using BioScript reverse transcriptase (BioLine, London, UK). Quantitation was achieved using SensiMix SYBR Low-Rox PCR mix (BioLine) with a 95°C 10 min hot start followed by 35 cycles of 95°C (10 s), 58°C (30 s) and 72° (30 s). Reverse transcription and water blanks were performed for each primer and sample. Primer sequences were: THTR-1 forward: 5′-ACCCCAGCTTCTAACCACCT-3′ and reverse: 5′-GGCGAGAGGAGTAGCACATC-3′; THTR-2 forward: 5′-CTGGCTCTGGTGGTCTTCTC-3′ and reverse: 5′-AGGCATAGCGTTCCACATTC-3′; Sp1 forward: 5′-TGCAGCAGAATTGAGTCACC-3′ and reverse: 5′-CACAACATACTGCCCACCAG-3′; β-actin forward: 5′-GGACTTCGAGCAAGAGATGG-3′ and reverse: 5′-CACAACATACTGCCCACCAG-3′. Results were quantified by the Pfaffl method [20] after determining primer efficiency separately using 10-fold dilutions of DNA spanning four orders of magnitude. Target gene mRNA was normalized to β-actin mRNA and expressed as the ratio to that of 5 mmol/l glucose culture control.

### Immunoblotting of thiamine transporters

Levels of thiamine transporter protein were determined by immunoblotting and chemiluminescent detection. hPTECs or HK-2 cells were rinsed with ice-cold PBS and harvested by scraping into lysis buffer (50 mmol/l tris-HCl pH 7.4, 5 mmol/l EDTA and protease inhibitor cocktail (PIC; Sigma, Poole, UK); 200 µl/75 cm^2^ cells). Cells were lysed by sonication and the membranes collected by sedimentation (30 min, 20,000 g, 4°C). Membranes were reconstituted in 200 µl resuspension buffer (50 mmol/l tris-HCl pH 7.4, 3 mmol/l MgCl_2_, 2% w/v SDS and protease inhibitor cocktail) mixed with 2× Laemmli buffer, boiled for 10 min, separated by 7.5% SDS-PAGE, transferred to PVDF membrane, blocked in 2% bovine serum albumin (w/v) in tris-buffered saline with 0.1% v/v Tween-20, probed with primary antibody, rinsed in tris-buffered saline with 0.1% v/v Tween-20, probed with secondary antibody, rinsed with tris-buffered saline with 0.1% v/v Tween-20 again and developed with ECL reagent. Exposed films were scanned using a calibrated scanner and densitometry calculations performed using ImageJ (http://rsb.info.nih.gov/ij/).

### Thiamine and phosphorylated metabolites and transketolase assay

Cellular concentrations of thiamine and its phosphorylated metabolites, thiamine monophosphate (TMP) and TPP, were determined by pre-column derivatization to thiochromes followed by separation by HPLC with fluorimetric detection [21]. The retention times, limits of detection and interbatch CV values for these metabolites were: thiamine 12.8 min, 32 fmol, 3.2; TMP 6.9 min, 6.7 fmol, 3.0; TPP 5.4 min, 94 fmol, 3.7. Results were expressed as pmol analyte/mg protein. Protein content was determined by the Bradford method.

Transketolase activity was measured using the enzymatic kinetic method of Chamberlain [22]. Briefly, washed cells were lysed by sonication in 10 mmol/l sodium phosphate buffer at pH 7.4. The lysate was diluted 5× then mixed with 200 µl assay buffer (final concentrations: 250 mmol/l tris-HCl pH 7.8, 14.8 mmol/l ribose-5-phosphate, 253 µmol/l NADH, 185 U/ml triosephosphate isomerase and 6 U/ml glycerol-3-phosphate dehydrogenase). 10 µl of either water or 8 mmol/l TPP was added to each sample and the absorbance at 340 nm and 37°C monitored for 2 hours. The rate of decline of absorbance was calibrated to an NADH standard curve and converted to enzymatic activity.

### Permeability of cell monolayers to thiamine

Primary hPTECs were seeded at 30–50% confluence onto polycarbonate filter transwell inserts of 12-well microplates. Each filter had surface area of 1.12 cm^2^ with 0.4 µm pores and 1×10^8^ pores cm^−1^ (cat no 3460, Corning, Amsterdam, Netherlands). hPTECs were grown to confluence in DMEM/F12 medium with 4 nmol/l thiamine and 5 mmol/l glucose and cultured for 7 days post-confluence before transfer to fresh medium for 5 days containing either 5 mmol/l or 26 mmol/l glucose with 4 nmol/l thiamine. Monolayer integrity was assessed after incubation with medium using Lucifer yellow dye [23]. [^3^H]Thiamine (specific activity 10 Ci/mmol) was purchased from American Radiolabelled Chemicals (St. Louis, MO, USA). For the determination of apparent permeability of thiamine (*P_App_*), medium containing 15 nmol/l [^3^H]thiamine was placed in either the apical or basolateral chamber (donor). Medium containing no thiamine was placed in the other (receiving) chamber and the monolayers were incubated for 1 h at 37°C with orbital shaking (120 rev/min) and medium from the chambers removed and counted. Radiotracer recovery from the chambers combined was >90%. Medium from the two chambers *P_App_* (cm.s^−1^) was deduced from the equation *P_App_* = (dQ/dT)×1/(A .C_0_) where dQ/dT is the steady state flux of thiamine across the cell monolayer membrane (µmol s^−1^) deduced from thiamine in the receiving medium, A the surface area of the hPTEC monolayer and C_0_ the initial thiamine concentration (µmolcm^−3^) in the donor chamber [23]. *P_App_* of control hPTEC monolayers was (8.3±2.3) ×10^−6^ cm.s^−1^ which is <1.7% of that of an empty filter, reflecting the tightness of the monolayer gap junctions and carrier-mediated transport of thiamine. The ratio of forward to reverse apparent permeability, *P_AppF/R_*, gives an indication of directional transport across the monolayers [23]; *P_AppF/R_* = 1, no net transport across the monolayer; *P_App_*_F/R_>1, net transport from the apical to basal face – reflecting active re-absorption of hPTECs; and *P_App_*_F/R_<1, net transport from the basal to the apical face – reflecting active excretion of hPTECs. The change in forward directional transport in high glucose concentration to low glucose concentration is given by
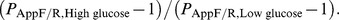



### Streptozotocin-induced diabetic rats

Male Sprague-Dawley rats, ∼300 g body weight, were purchased from Charles River UK Ltd (Ramsgate, Kent, U.K.). They were kept 2 per cage at 21°C, 50–80% humidity and with daily 12 h light cycle, and had free access to food and water. Diabetes was induced with i.v. injection of STZ (55 mg/kg) in 50 mM sodium citrate buffer, pH 4.5. Control animals received vehicle alone. Diabetic rats were randomised to 2 study groups: diabetic control (DC) – maintained with diabetes for 12 weeks; and DIT – maintained with diabetes for 6 weeks followed by intensive insulin therapy. Insulin (s.c., o.i.d) was administered 4 U per day to diabetic rats to moderate hyperglycemia and maintain body weight. DIT rats received insulin implants (LinShin Canada, Inc. Toronto, Canada) with sustained release of 6 U insulin per day for 6 weeks. Rats were maintained for 12 weeks. Animal studies were performed on UK Home Office project license no 40/3261. The study was approved by the University of Warwick Biological Ethics Sub-Committee. Plasma glucose, glycated hemoglobin HbA_1_, and plasma and urinary thiamine were determined as previously described. Creatinine concentrations in plasma and urine were determined by LC-MS/MS [24]. Renal clearance of thiamine and fractional excretion of thiamine were deduced.

### Statistical analysis

Data are shown as mean ± SD or median (lower – upper quartile). Significance of differences of means was deduced by two-tailed Student's *t* test and of medians by Mann Whitney-U test. Correlation analysis was by the Spearman non-parametric method.

## Results

### High expression of thiamine transporters in the human kidney is localised to the tubular epithelium

**Figure 1:**
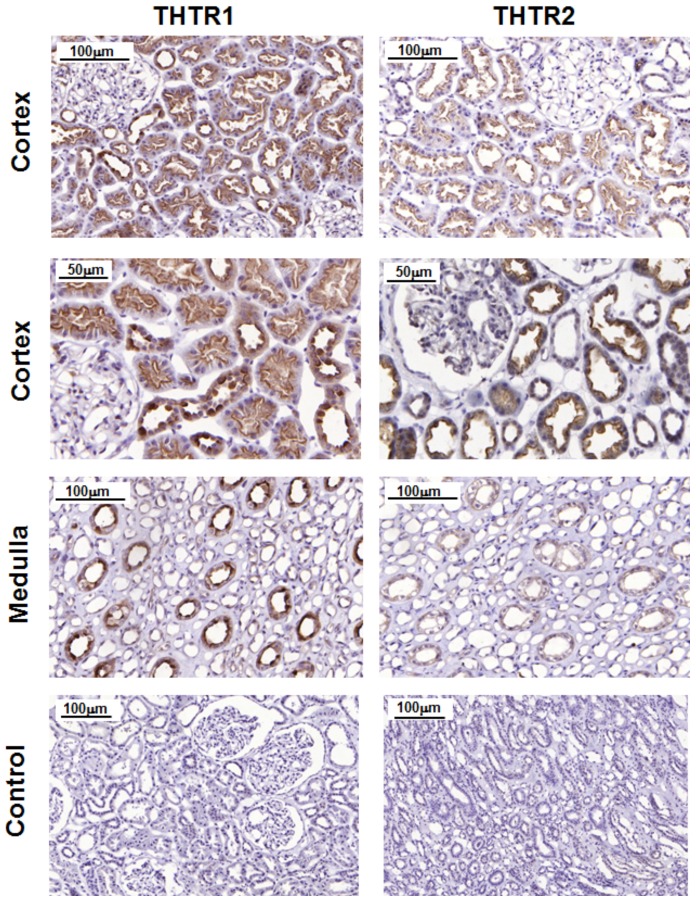
Protein expression of thiamine transporters in human kidney, immunostaining for THTR-1 and THTR-2. Cortex - THTR-1: Intense polarised staining of the apical, luminal membranes in proximal tubules. THTR-2: similar staining to THTR-1 in proximal tubules. Distal tubules - Cellular staining throughout, stronger for THTR-1 than THTR-2 protein. Glomeruli: no staining for both proteins. Medulla - uniform, diffuse staining throughout cells of the collecting duct for THTR-1 and THTR-2, with much stronger staining for THTR-1. Image shows brown 3,3′-diaminobenzidine stain with blue haematoxylin-counterstaining of nuclei. Control tissue: omission of primary antibodies.

Tissue sections from five normal human kidneys were stained for thiamine transporter location using immunohistochemistry. Typical staining patterns for THTR-1 and THTR-2 in sections of normal cortex and medulla are shown (Figure 1). In the cortex there was intense polarised staining of the apical, luminal membranes in proximal tubules for THTR-1 and THTR-2. There was no staining for both proteins in glomeruli. In the medulla staining for THTR-1 and THTR-2 was much weaker than in the cortex. There was uniform, diffuse staining throughout cells of the collecting duct for THTR-1 and THTR-2, with stronger staining for THTR-1.

### Profound decrease of thiamine transporter expression in human proximal tubular epithelial cells in primary culture by high glucose concentration and impaired thiamine monolayer permeability

**Figure 2:**
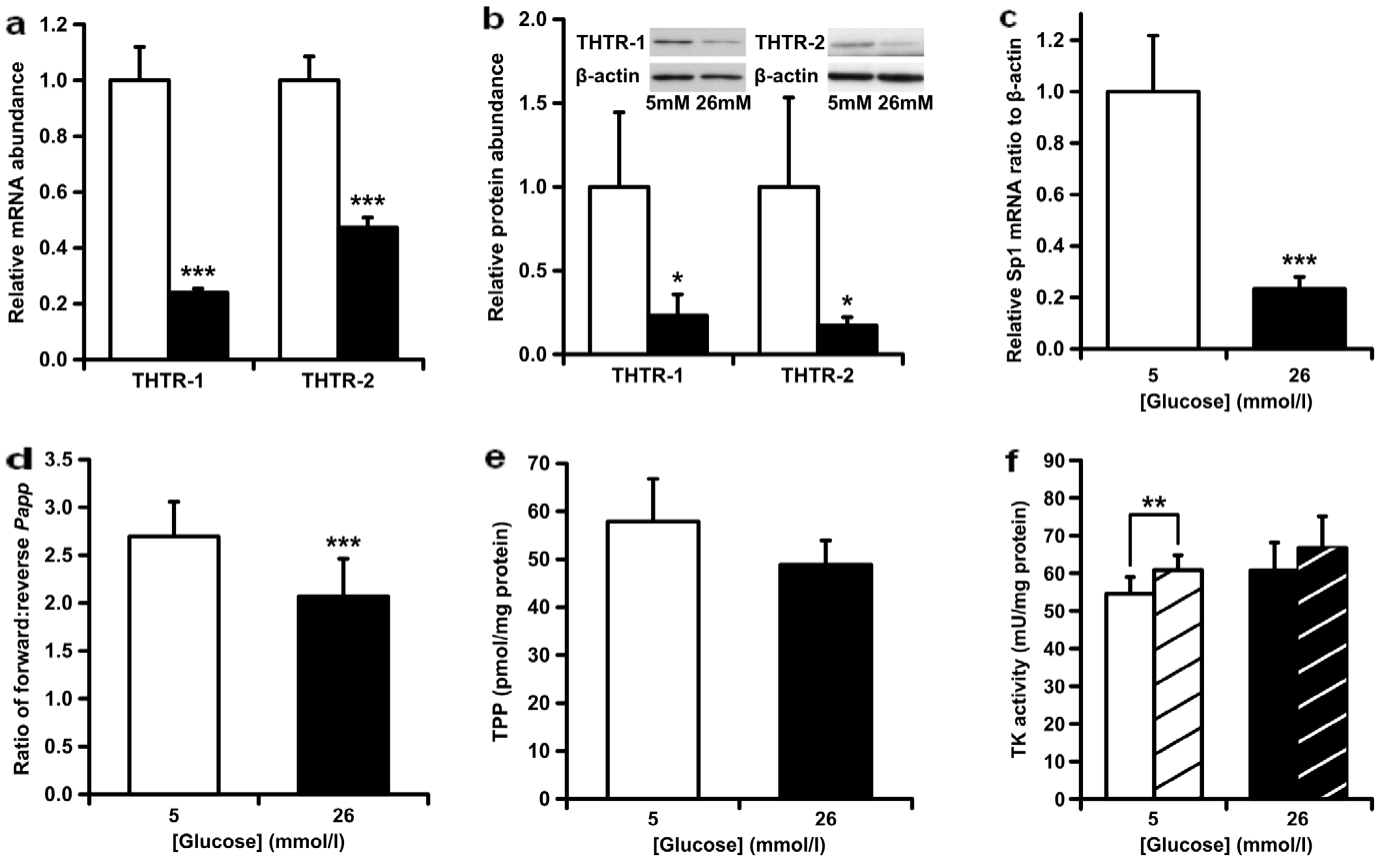
Effect of glucose concentration on thiamine-related metabolism in human tubular epithelial cells in primary culture. **a** mRNA of *SLC19A2* and *SLC19A3* genes, relative to β-actin (n = 8). **b** THTR-1 and THTR-2 protein in membrane extracts, relative to β-actin (n = 4). **c** Effect of glucose concentration on expression of transcription factor Sp1. Sp1 mRNA level was normalized to β-actin mRNA. Data are mean ± SD (n = 8). **d** Ratio of forward to reverse thiamine apparent permeability *P_AppF/R_*. **e** Cellular concentration of TPP. **f** Transketolase activity. Key: hPTECs in primary culture for 5 days with 5 mmol/l glucose (hollow bars), 26 mmol/l glucose (filled bars), and addition of 350 µmol/l TPP in the TK activity assay (striped bars). Data are mean ± SD (n = 8, 4, 8, 6, 8 and 8 for **a**–**f**, respectively). Significance: *, *P*<0.05; **, *P*<0.01 and *** *P*<0.001.

hPTECs were isolated from kidney cortex of healthy human donors. They were identified by characteristic morphology, absence of staining for uromodulin and intense and uniform staining for aquaporin 1. When hPTECS were incubated in medium containing a high glucose concentration (26 mmol/l) for 5 days the expression of thiamine transporters, as judged by levels of *SLC19A2* and *SLC19A3* mRNA, was markedly decreased with respect to hPTECs incubated in medium containing 5 mmol/l glucose. Changes in mRNA were: *SLC19A2*, −76% (*P*<0.001), and *SLC19A3*, −53% (*P*<0.001) - Figure 2A. The levels of THTR1 and THTR2 proteins were investigated by Western blotting of membrane extracts of hPTECs cultured under the same conditions. THTR-1 and THTR-2 protein bands were detected at 60 and 58 kDa, respectively. Band specificity was indicated by only one band of molecular mass similar to predicted molecular mass, 55.4 and 55.6 kDa, respectively. THTR-1 and THTR-2 protein levels were markedly decreased in hPTECs incubated in high glucose concentration with respect to low glucose concentration containing control. Changes in thiamine transporter proteins were: THTR-1, −77%; THTR-2, −83% (*P*<0.05) - Figure 2B.

Transcription factor Sp1 is involved in regulation of the expression of both *SLC19A2* and *SLC19A3* genes. The expression of Sp1 in hPTECs in primary culture and effect of glucose concentration was investigated and was found to be decreased 77% hPTECs in 26 mmol/l glucose, with respect to cultures grown in 5 mmol/l glucose - as judged by decrease of Sp1 mRNA (Figure 2C).

The effect of change in thiamine transporter expression on the permeability of confluent monolayers of hPTECs to thiamine was investigated. Incubation of hPTECs in 26 mmol/l glucose for 5 days produced a decrease in active transport of thiamine across the cell monolayer. *P_App_*_F/R_ for [^3^H]thiamine in hPTEC monolayers decreased 23% with respect to *P_App_*_F/R_ for monolayers cultured in 5 mmol/l glucose (decreased from 2.70 to 2.07, *P*<0.001, Figure 2D). This represents a decrease in apical to basolateral directional transport across PTEC monolayers of 37%.

The effect of decreased transporter protein and decreased directional transport of thiamine by hPTECs in high glucose concentration on cellular metabolites of thiamine was investigated. There was no significant change of cellular concentration of TPP in hPTECs incubated with 4 nmol/l thiamine and 26 mmol/l glucose compared with 4 nmol/l thiamine and 5 mmol/l glucose control cultures (Figure 2E). The concentrations of both thiamine and TMP were below the limit of detection (<0.15 and 0.03 pmol/mg protein respectively; <0.3% total thiamine metabolites). Concomitant with the lack of difference in intracellular TPP concentration, there was no change in the activity of the TPP-dependent enzyme, transketolase. A low “thiamine-effect”, or unsaturation of transketolase, of 11% was observed in the 5 mmol/l glucose cultures with no significant thiamine effect present in the 26 mmol/l glucose cultures (Figure 2F).

### Thiamine transporter expression and thiamine metabolism are markedly different in human proximal tubular epithelial cells in primary culture compared to the human HK-2 cell line

**Figure 3:**
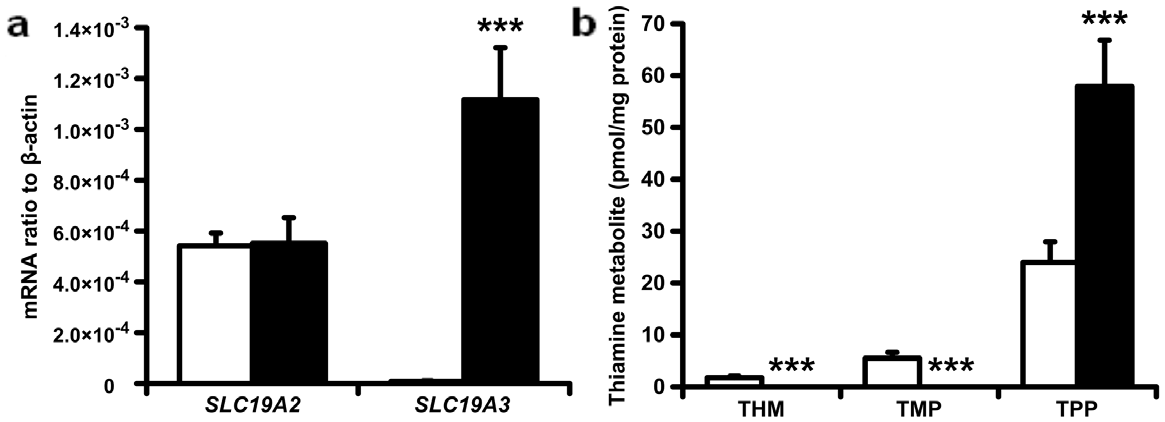
Comparison of thiamine transporter expression and thiamine metabolites in human kidney cells and HK-2 cell line. **a**. *SLC19A2* and *SLC19A3* mRNA. Key: HK-2 cells (hollow bars) and human kidney cortex (filled bars). The bar for *SLC19A3* mRNA in HK-2 cells is 122-fold lower than for human kidney cortex. **b**. Cellular metabolites of thiamine. Key: HK-2 cells (hollow bars) and hPETCs (filled bars). Thiamine and TMP in hPTECs were lower than the limits of detection. Data are mean ± SD (n = 4 and 8 for **a** and **b** respectively). Significance: ***, *P*<0.001 (with respect to HK-2 cells).

The HK-2 cell line, produced by human papilloma virus [25], is often used as a model for studies of hPTECs. We compared thiamine transporter expression and metabolism of HK-2 cells and hPTECs in primary culture. There were marked difference - the most striking of which was the almost complete absence of THTR-2 expression in the HK-2 cells, both at the level of mRNA (Figure 3A) and protein (data not shown). Furthermore, total intracellular thiamine metabolite concentration (thiamine+TMP+TPP) was higher in primary hPTECs than in HK-2 cells for culture in 5 mmol/l glucose and 4 nmol/l thiamine (57.9±8.9 versus 31.3±4.1 pmol/mg protein; *P*<0.01). The thiamine pool of hPTECs was exclusively in the form of TPP whereas the thiamine pool of HK-2 cells contained significant proportions of thiamine and TMP (18% and 6% respectively; *P*<0.001) (Figure 3B).

### Intensification of glycemic control improves renal clearance of thiamine in experimental diabetes

To gain further evidence for a link of renal clearance of thiamine and glycemic control in diabetes *in vivo* we studied the effect of intensive glycemic control on established increased renal clearance of thiamine in experimental diabetes. STZ-induced diabetic rats had body weight maintained and hyperglycemia moderated by daily insulin injections for 6 weeks. Thereafter they were randomised to two groups: diabetic control and diabetic with intensive insulin therapy for a further 6 weeks where the latter group received intensive therapy by insulin implants. Diabetic status was assessed by plasma glucose concentration and glycated hemoglobin HbA_1_. Renal thiamine mishandling was assessed by renal clearance of thiamine and fractional excretion of thiamine. After 12 weeks, HbA_1_ was increased in diabetic controls but not increased significantly in the intensive insulin therapy group, reflecting improved glycemic control in the latter group. Clearance of thiamine increased 2-fold and fractional excretion of thiamine 3-fold in diabetic controls, with respect to healthy rats (*P*<0.01). Intensive insulin therapy reversed this increase in fractional excretion of thiamine by 77% (*P*<0.001). For the diabetic groups combined, HbA_1_ correlated positively with thiamine clearance (r = 0.64, *P*<0.05; Spearman) and thiamine fractional excretion (r = 0.68, *P*<0.01) of thiamine. This indicates that intensive insulin therapy attenuates but does not correct renal thiamine mishandling in streptozotocin-induced diabetic rats (Table 1).

**Table 1:** Physiological and biochemical characteristics of streptozotocin-induced diabetic rats and healthy controls.

Rat study group	Healthy controls	Diabetic control	Diabetic with insulin intensive therapy
n	8	7	8
Body weight (g)			
Baseline	328±29	330±11	322±19
6 weeks	500±27	302±33***	307±24***
12 weeks	590±37	310±42***	478±246*** ^OOO^
HbA_1_ (%)	5.30±1.85	7.03±0.64*	5.94±1.34
Urinary albumin excretion (mg/24 h)	0.10 (0.07–0.12)	2.48 (1.76–3.21)**	0.70 (0.33–1.60)**
Renal clearance of thiamine (ml/min)	0.92±0.23	1.57±0.32**	1.36±0.56
Fraction excretion of thiamine (%)	28.1±9.0	80.6±17.0***	40.2±17.2^OOO^
Thiamine reuptake (% filtered)	69 (64–80)	16 (6–28)***	56 (45–79)^OO^
Food consumption (g/24 h)	21.9±2.2	49.8±10.8***	40.3±7.3***
Thiamine consumption (mg/24 h)	0.68±0.07	1.55±0.33***	1.25±0.23***

## Discussion

In this work we found that high glucose concentration decreases thiamine transporter expression in hPTECs and impairs apical to basolateral thiamine transport, and intensification of glycemic control improves established increased renal washout of thiamine in experimental diabetes.

**Figure 4:**
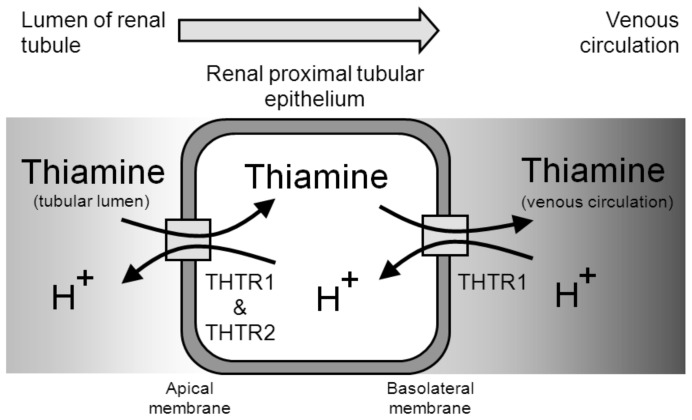
Schematic diagram of thiamine transpermeability across the proximal tubular epithelium.

Immunohistochemical staining of normal human kidney sections showed intense staining for THTR-1 and THTR-2 in the proximal tubule indicating that the proximal tubule is the likely major site of renal thiamine reuptake in the kidney. There was polarization of both THTR-1 and THTR-2 to the apical membranes of hPTECs. Previous studies have detected THTR-1 protein in both basolateral and apical membranes of the proximal tubular epithelium and THTR-2 protein only in apical membranes [26], [27]. Therefore, thiamine likely crosses the human proximal tubular epithelium by THTR-1 and THTR-2 mediated uptake on the apical side and efflux on the basolateral side mainly by THTR-1 (Figure 4).

Studies with hPTECs in primary culture incubated with high glucose concentration showed decreased expression and abundance of both thiamine transporters with a concomitant decrease in net thiamine transport from the apical to the basolateral sides of hPTEC monolayers, with respect to 5 mmol/l glucose control cultures. The decrease in transpermeability of thiamine across hPTEC monolayers in high glucose concentration – change in ratio of forward and reverse monolayer transport (P_AppF/R_ ratio), 23%, was small compared to decrease in total thiamine transporter protein - THTR-1, 77%, and THTR-2, 83%. This may be due to limited polarized decrease in thiamine transporter expression and activity – for example, greater decline in THTR-1 and THTR-2 protein and activity on the apical surface than the basolateral surface of hPTEC monolayers - required to change the balance between forward and reverse apical to basolateral flow in hPTEC monolayers.

The decrease in transpermeability of thiamine across hPTEC monolayers was, however, relatively small compared to the marked apparent decrease in tubular re-uptake of thiamine in experimental diabetes. Tubular re-uptake of thiamine was deduced from the amount of thiamine filtered in the glomeruli (GFR x [thiamine]_plasma_) minus the amount of thiamine excreted in urine. In STZ-induced diabetic rats there was no detectable tubular re-uptake of thiamine [7] and in clinical diabetes tubular re-uptake of thiamine decreased from 7.7 µmol per 24 h in healthy people to 0.3 µmol per 24 h in patients with diabetes (−96%) [3]. The volume of distribution (V_D_) of thiamine in human subjects estimated from pharmacokinetics of thiamine is 16.7 l [28]. For normal GFR, it can be concluded that the free thiamine pool passes through the proximal tubules at least 10 times per 24 h. If 37% thiamine is lost on each pass, the effective decrease in thiamine re-uptake per 24 h is 95% (residual fractional uptake of thiamine = 0.63^10^ or 1%). Hence renal clearance of thiamine is very sensitive to change in tubular thiamine re-uptake. In diabetes there is also progressive functional decline of the proximal tubular epithelium [29].

The mechanism of decreased expression of the thiamine transporters in hPTECs in model hyperglycemia is unknown may be linked to transcription factor Sp1. Incubation of hPTECs in high glucose concentration produced decreased Sp1 mRNA. Decreased Sp1 expression was found previously in vascular endothelial cells incubated in high glucose concentration *in vitro* and diabetes *in vivo* [30]. Increased O-glycosylation of Sp1 protein by activation of the hexosamine pathway is implicated in down regulation of Sp1-dependent gene expression in renal mesangial cells exposed to high glucose concentration [31] but not in proximal tubular epithelial cells. We examined hexosamine pathway activation in hPTECs incubated in high glucose concentration by assay of uridine diphosphate-N -acetylglucosamine and uridine diphosphate-glucose [32] but found no evidence of activation – data not shown.

The HK-2 cell line exhibited markedly different thiamine metabolism to hPTECs in primary culture and so was not used further. THTR-2 is a key component of thiamine handling *in vivo* [17], [33] and this has markedly low expression in HK-2 cells. Levels of cellular thiamine metabolites were also markedly different in HK-2 cells compared to those of hPTECs in primary culture. This may be linked to the use of supra-physiological micromolar concentrations of thiamine in commercially available tissue culture medium. For thiamine transporter expression we found *SLC19A2*∶*SLC19A3* mRNA ratios of 1∶2 and 60∶1 respectively for hPTECs and the HK-2 cell line.

Once across the tubular epithelium, thiamine crosses the vascular endothelium to enter capillary lumen and venous circulation. There is generally paracellular permeability in the vascular endothelium, through 4–7 nm diameter pores of gap junctions, and hence this likely does not present a barrier for reabsorption into the venous circulation [34]; paracellular permeability is available for thiamine since it has mean molecular diameter of *ca.* 1.4 nm [35].

We conclude that high glucose concentration decreased expression and activity of the thiamine transporters THTR-1 and THTR-2 in hPTECs in primary culture, producing a concomitant decrease in apical to basolateral transport. Together with evidence that intensification of glycemic control corrected increased clearance of thiamine in experimental diabetes, we conclude that there is hyperglycemia-induced mishandling of thiamine in the kidney in diabetes linked to decreased expression of thiamine transporters in the renal tubular epithelium. Hyperglycemia-induced thiamine transporter dysregulation in the tubular epithelium causes increased renal clearance of thiamine – a novel type of renal washout of thiamine linked to thiamine insufficiency previously only linked to diuretic-induced increased diuresis [36]. Hyperglycemia-induced thiamine transporter dysregulation likely explains the increased renal washout of thiamine in clinical diabetes.
